# Trait self-responsibility modulates neural responses to near-miss loss: an ERP study

**DOI:** 10.3389/fnhum.2026.1798837

**Published:** 2026-03-03

**Authors:** Xin Jin, Hanmo Yin

**Affiliations:** 1 Institute of Education and Society, University College London, London, United Kingdom; 2 Centre for Research in Psychology and Human Well-Being (PsiTra), Faculty of Social Science and Humanities (FSSK), Universiti Kebangsaan Malaysia, Bangi, Malaysia; 3 Psychology Program, Faculty of Social Science and Humanities (FSSK), Universiti Kebangsaan Malaysia, Bangi, Malaysia

**Keywords:** ERP, FRN, near-miss effect, P300, trait self-responsibility

## Abstract

**Introduction:**

Near-miss loss is outcome that is objectively loss but physically proximal to a win, often triggering higher physiological arousal and motivation than regular loss (full loss). This study investigated how trait self-responsibility modulates the behavioral and neural processing of near and full gambling outcomes.

**Methods:**

Participants were categorized into high and low trait self-responsibility groups, performed a “Wheel of Fortune” gambling task while electroencephalography (EEG) was recorded.

**Results:**

Behavioral results showed that while near-miss loss universally induced counterfactual thinking, the high trait self-responsibility group reported significantly higher pleasantness and sustained gambling motivation compared to the low trait self-responsibility group. Electrophysiologically, the Feedback-Related Negativity (FRN) showed no group differences, suggesting that early outcome valuation is insensitive to personality traits. However, the P300 component revealed a significant interaction: the high trait self-responsibility group exhibited attenuated P300 amplitudes specifically for near-miss losses compared to full losses.

**Discussion:**

These findings indicate that trait self-responsibility modulates the near-miss effect at the late cognitive stage, where the increased internal processing load in high trait self-responsibility individuals likely competes with the attentional resources allocated to external feedback.

## 1 Introduction

A near-miss loss is formally a loss but physically proximal to a win, which represents a unique outcome (Reid, 1986). Although objectively equivalent to full loss (i.e., zero monetary gain), near-miss loss is psychologically perceived as a “close call” rather than total failure. Extensive behavioral research indicates that, compared to full losses, near-miss loss elicits heightened physiological arousal and more negative affective states (Dixon et al., 2013; Qi et al., 2011; Griffiths, 1991), yet paradoxically induce a stronger motivation to continue gambling (Clark et al., 2009; Reid, 1986). This phenomenon is widely known as the “near-miss effect.”

Electroencephalography (EEG) has been widely utilized to examine the cognitive characteristics of the near-miss effect, with two event-related potential (ERP) components commonly analyzed: feedback-related negativity (FRN) and the P300. The FRN is a negative deflection recorded from frontal-central recording scalp sites, peaking approximately 250–300 ms following feedback onset (Miltner et al., 1997; Gehring and Willoughby, 2002). It is thought to reflect negative reward prediction errors (Holroyd and Coles, 2002; Sambrook and Goslin, 2015). The P300 is characterized by a positive deflection with a central-parietal maximum, typically occurring approximately 300–600 ms after stimulus onset. Its amplitude is widely recognized as an index of attentional resource allocation, sensitive to factors such as subjective probability, motivational significance, and the level of attentional engagement (Nieuwenhuis et al., 2005). Extensive research has examined the electrophysiological dissociation between near-miss loss and full loss, yet findings regarding these key ERP components remain inconsistent. For the P300, while some studies have reported larger amplitudes for near-miss losses compared to full losses (Alicart et al., 2015; Qi et al., 2011), others have observed the opposite pattern, with full losses elicited greater P300 responses (Kreussel et al., 2013; Ulrich and Hewig, 2014). Furthermore, several studies found no significant P300 differences between the two outcomes (Lole et al., 2013; Luo et al., 2011). A similar lack of consensus exists regarding the FRN. Some researchers have reported a larger (more negative) FRN following near-miss losses (Kreussel et al., 2013; Ulrich and Hewig, 2014), whereas others found a smaller FRN (Luo et al., 2011) or no significant distinction at all (Habib and Dixon, 2010; Qi et al., 2011).

These inconsistencies underscore a critical question regarding the modulating factors and boundary conditions of the near-miss effect. Recent research has identified several contextual determinants. For instance, critical factors include the stopping position of the reel (whether the near-miss loss stops before or after the payline; Dores et al., 2020) and the reward expectancy (the proportion of the winning area; Yin et al., 2024). These factors have been shown to significantly influence the relative ERP amplitudes of near-miss loss versus full loss, effectively determining the direction of the difference for P300 and FRN components (i.e., which outcome elicits a larger response).

However, another important facet remains elusive: establishing stable differences in the neural correlates of the near-miss effect across distinct experimental groups has been proven difficult. For example, Xia et al. (2018) investigated whether susceptibility to near-miss loss differed between optimists and pessimists. While they observed distinct behavioral patterns—with optimists exhibiting riskier betting behavior following near-miss loss—they found no significant group differences in ERP mean amplitudes. This dissociation between behavioral susceptibility and neural responsiveness highlights the challenge of identifying stable electrophysiological markers across different personality traits. Similarly, studies focusing on pathological traits have encountered comparable challenges. Ulrich and Hewig (2018) compared problem gamblers with matched controls and found that the specific neural processing of near-miss outcomes (reflected by P300 and FRN) did not differ between the two groups. These findings imply that such broad traits may lack the sensitivity to index the fundamental electrophysiological mechanisms of the near-miss effect. Consequently, our understanding of how individual differences modulate near-miss processing remains limited. Therefore, it is necessary to examine traits that are more directly related to the structural features of the near-miss effect.

Beyond electrophysiological responses, behavioral research has delineated the near-miss effect as a multifaceted psychological phenomenon involving distinct cognitive, affective, and motivational processes. Consequently, specific subjective measures were selected to capture these dimensions. Counterfactual thinking serves as a cognitive index of the proximity to the win, reflecting mental simulations of alternative outcomes (Kahneman and Varey, 1990; Roese, 1997). Pleasantness ratings assess the immediate affective valuation, capturing the paradoxical negative valence and frustration often associated with near-miss loss (Dixon et al., 2013; Qi et al., 2011). Reward anticipation reflects an illusionary thought that near-miss loss is a predictor of future success rather than random failure (Langer, 1975; Wohl and Enzle, 2002). Finally, Gambling motivation quantifies the behavioral urge to persist, representing the functional consequence of the near-miss signal (Clark et al., 2009; Reid, 1986).

However, historical empirical findings reveal that the specific emotional or cognitive responses triggered by near-miss loss do not manifest consistently, rendering the nature of the near-miss effect elusive. For example, some studies found that near-miss loss elicited more negative emotion (Qi et al., 2011; Clark et al., 2012) or stronger gambling motivation (Clark et al., 2009) than full loss, while some suggested such dissociation did not exist (Ulrich and Hewig, 2014). Crucially, these studies did not account for the role of individual differences in modulating the perception of gambling outcomes. Therefore, integrating these measures allows us to dissect how specific individual traits shape the interpretative framework of the near-miss effect.

To deeply explore the processing of gambling outcome, particularly near-miss loss, through the lens of individual differences, it is instructive to revisit the fundamental theoretical frameworks of the near-miss effect. A core theory explaining the near-miss effect is the “illusion of control” (Langer, 1975). Researchers argue that a near-miss loss transmits a spurious signal of skill acquisition, reinforcing the perception that victory is imminent (Griffiths, 1991). Notably, the perception of this skill signal relies heavily on how individuals attribute outcomes (i.e., whether they perceive the outcome as contingent upon their own actions). Empirical studies have substantiated the role of personal agency in modulating the near-miss effect. For instance, Clark et al. (2012) demonstrated that near-miss outcomes elicited significantly greater physiological arousal (e.g., electrodermal activity) and subjective motivation to continue playing specifically when participants actively selected their gambles. In contrast, when outcomes were computer-generated (thereby minimizing personal responsibility), the distinct impact of near-miss loss was markedly diminished. This suggests that the provision of choice serves as a catalyst, transforming the near-miss loss from a random “non-win” into a salient feedback signal of “almost succeeding,” which in turn triggers more intensive cognitive and emotional processing. However, this line of study has predominantly focused on transient, situational manipulations of agency, largely overlooking the regulatory role of stable personality traits, specifically trait self-responsibility (TSR).

Self-responsibility refers to a stable psychological disposition wherein individuals hold themselves accountable for their actions and consequences (Zheng, 2009). Grounded in locus of control theory, individuals with high self-responsibility tend to make internal attributions, viewing outcomes as reflections of their own effort or ability rather than luck (Rotter, 1966). In the context of near-miss loss, this trait may act as a “double-edged sword.” While low trait self-responsibility individuals may dismiss a near-miss loss as mere bad luck, high trait self-responsibility individuals are more likely to interpret it as an “operational error” or meaningful feedback on their skill. Consequently, to resolve the cognitive dissonance caused by this “error,” these individuals may experience a stronger compulsion to gamble again to “correct” the outcome (Amsel, 1958; Stange et al., 2016).

In summary, although the neural mechanisms underlying the near-miss effect have been explored, the specific modulatory role of trait self-responsibility, as a core variable influencing outcome attribution and performance monitoring, remains unknown. The present study utilized the Self-Responsibility Questionnaire for College Students (Zheng, 2009) to screen participants with high and low trait self-responsibility. Participants performed a gambling task while their EEG was recorded, and subsequently rated their subjective experiences regarding counterfactual thinking, pleasantness, gambling motivation, reward anticipation for the next trial, and perceived responsibility across different outcomes. We hypothesize that trait self-responsibility modulates the processing of near-miss outcomes. Specifically, we hypothesize that individuals with different levels of trait self-responsibility will exhibit differential behavioral and ERP patterns when processing near-miss loss versus full outcomes, accompanied by differences in perceived responsibility levels in the gambling task.

## 2 Method

### 2.1 Participants

The college students’ trait self-responsibility questionnaire developed by Zheng (2009) was administered to a random sample of 450 university students. A total of 386 valid questionnaires were returned, yielding a response rate of 85.78%.

The questionnaire consists of 34 items across three dimensions: Self-Responsibility Cognition, Self-Responsibility Affect, and Self-Responsibility Behavior. These dimensions are further divided into nine specific factors: Morality, Planning, and Attribution; Initiative and Independence; and Aversion, Self-blame, Adjustment, and Satisfaction. The items are rated on a 5-point Likert scale ranging from 1 (“Very inconsistent”) to 5 (“Very consistent”). Higher scores indicate a higher level of trait self-responsibility.’

The internal consistency coefficients (Cronbach’s *α*) for the first-order factors ranged from 0.469 to 0.680, and split-half reliability coefficients ranged from 0.358 to 0.660. For the second-order dimensions, internal consistency coefficients ranged from 0.731 to 0.771, with split-half coefficients between 0.622 and 0.746.

Based on the total scores, participants were ranked. The top 20 participants with the highest scores and the bottom 20 participants with the lowest scores were selected to form the high trait self-responsibility group (High-TSR) and the low trait self-responsibility group (Low-TRS), respectively, for the subsequent ERP experiment.

Ten participants were excluded due to incomplete experimental procedures or excessive artifacts. Consequently, 30 participants were included in the final statistical analysis. The High-TSP group consisted of 15 participants (6 males; M_age_ = 18.87 ± 0.64) with a mean questionnaire score of 157.40 ± 4.50. The Low-TRS group consisted of 15 participants (6 males; M_Age_ = 18.87 ± 1.12) with a mean score of 108.66 ± 16.05. An independent samples *t*-test revealed a significant difference in scores between the two groups [*t*(28) = 11.32, *p* < 0.001], confirming the effectiveness of the participant screening process.

All participants provided written informed consent. The study received ethical approval from the local ethics committee. All procedures were conducted in accordance with the relevant guidelines and regulations, including the Declaration of Helsinki.

### 2.2 Procedure and task

Participants were seated comfortably in a dimly lit, sound-attenuated chamber after being fitted with the electrode cap. They viewed the monitor from a distance of approximately 100 cm. The screen background was gray, and the stimuli were presented within a visual angle of less than 5° both horizontally and vertically. Participants were instructed that they would be participating in a rapid-reaction mini-game. They were initially endowed with a base fund of 30 CNY. Participants were informed that any winnings during the game would be added to this base fund, while any losses would be deducted from it. Upon completion of the experiment, participants would receive cash remuneration based on their cumulative results and performance.

**Figure 1:**
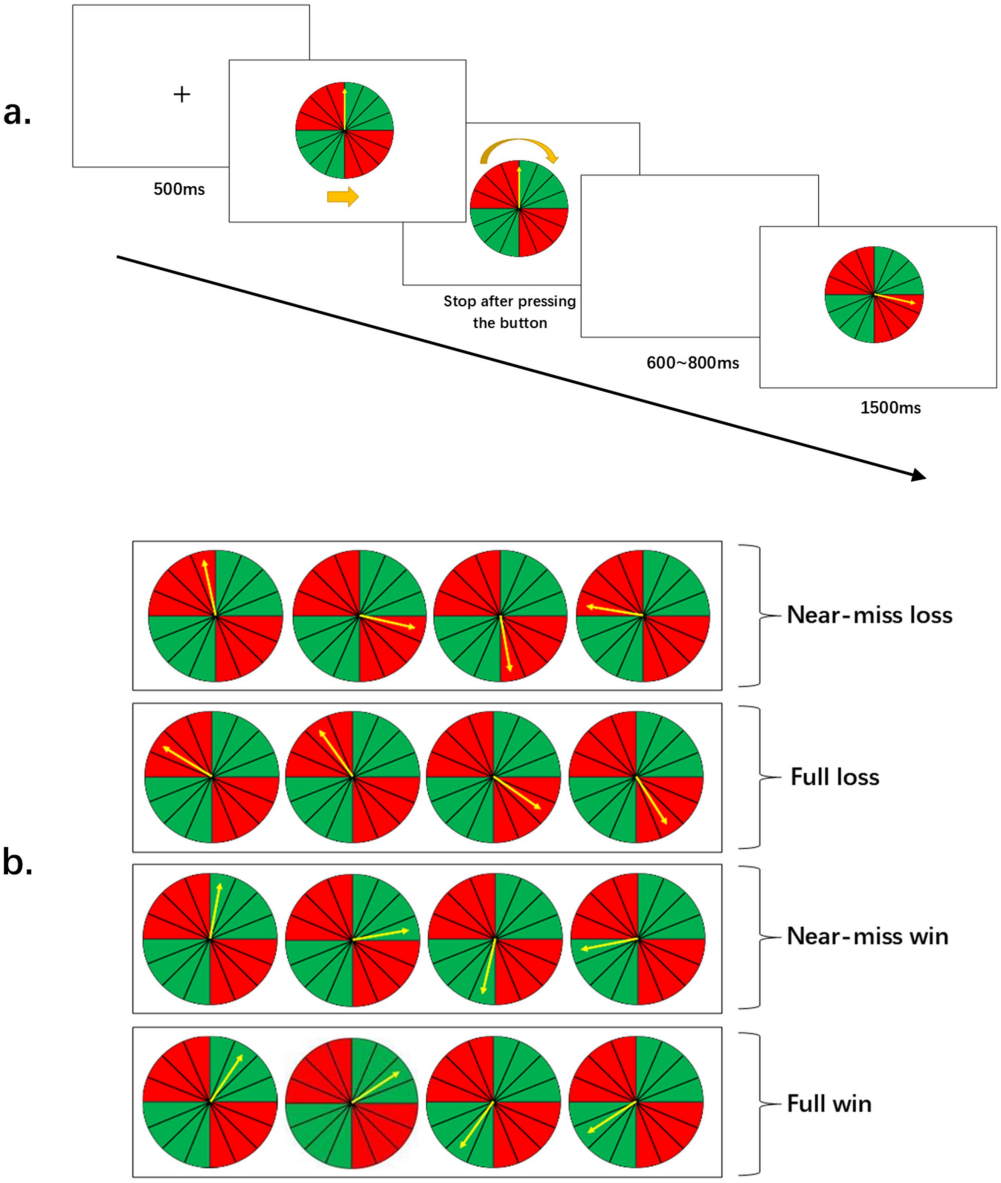
Schematic representation of the experimental task and outcome conditions. **(a)** Timeline of a single trial. **(b)** Illustration of the four outcome categories based on the stopping position of the pointer relative to the sector boundaries.

The experimental task was adapted from the wheel of fortune task by Ulrich and Hewig (2014), as illustrated in Figure 1a. At the onset of each trial, a fixation cross “+” appeared on the screen for 500 ms. Subsequently, a wheel and a vertical yellow pointer appeared in the center of the screen. The wheel was uniformly divided into 16 sectors, alternating between red and green in groups of four. When the participant pressed the spacebar, the pointer began to rotate rapidly (with an equal number of clockwise and counter-clockwise trials).

Participants were informed that upon pressing a key rapidly, the yellow pointer would immediately stop at the sector where it was currently traversing. Their task was to maximize winnings and avoid losses. Feedback outcomes were presented in a pseudo-random manner. The pointer’s rotation speed was adjusted to be sufficiently fast, preventing participants from accurately judging if the stopping position was linked to their reaction (Suo et al., 2009; Wu et al., 2017). To ensure the reliability of the experimental design, we conducted a debrief after the experiment to ask if they felt the results were actually random instead of under their control. No participants reported suspecting that the outcomes were rigged, which indicates that both groups remained unaware that the stopping position was predetermined.

Upon pressing the spacebar a second time, followed by a random blank interval of 600–800 ms, the pointer stopped and remained on a specific sector for 1,500 ms. If the pointer landed on the green sector, it indicated a financial gain; if it landed on the red sector, it indicated a financial loss. The magnitude of each gain or loss was 1 CNY. The task then proceeded to the next trial. At the end of each block, the cumulative amount of winnings was presented to the participants.

As illustrated in Figure 1b, feedback outcomes were categorized into four types based on the color and spatial distribution of the sectors: near-miss loss, full loss, near-miss win, and full win. The entire experimental procedure was implemented and controlled using E-Prime 2.0. The experiment consisted of 4 blocks, with each block containing 96 trials, for a total of 384 trials. Each of the four trial types occurred 96 times.

Participants were allowed a self-paced break of 3–5 min between blocks. The formal experiment lasted approximately 30 min. Prior to the formal experiment, participants completed a practice session consisting of 20 trials (identical to the formal task) to ensure familiarity with the experimental procedure.

Upon completion of the experiment, participants rated their feelings about different outcomes (near-miss loss, full loss, near-miss win, full win) using a 7-point Likert scale (1 = not at all, 7 = strongly): (1) Counterfactual thinking (“*I thought that if I had pressed the button a little faster or slower, I would have won.*”); (2) Pleasantness (“*I felt pleased when I saw this outcome.*”); (3) Gambling motivation (“*I had the urge to immediately proceed to the next trial*.”); (4) Reward anticipation for the next trial (“*I thought I would win money in the next round*.”); (5) Perceived responsibility (“*I felt responsible for the result*.”). The Perceived responsibility item is manipulation check to test whether participants in the high trait self-responsibility group indeed perceived a significantly higher level of self-responsibility.

### 2.3 EEG recordings and quantification

EEG data were recorded and analyzed using a Neuroscan system. The EEG was recorded using a 32-channel electrode cap with reference electrodes placed on the bilateral mastoids and a ground electrode located at the midpoint between FPz and Fz. Horizontal (HEOG) and vertical electrooculograms (VEOG) were recorded simultaneously. Electrode impedances were maintained below 5 kΩ. The data were digitized at a sampling rate of 500 Hz with a band-pass filter of 0.05–100 Hz. For offline analysis, the EEG data were segmented into epochs ranging from 200 ms pre-feedback (serving as the baseline) to 1,000 ms post-feedback. The onset of feedback was defined as the moment the yellow pointer stopped on a sector. The data were digitally filtered with a zero-phase band-pass filter of 0.1–30 Hz and baseline-corrected using the mean amplitude of the pre-feedback interval. Ocular artifacts were corrected automatically. Trials containing artifacts with amplitudes exceeding ±80 μV were automatically rejected prior to averaging. After artifact rejection, the number of valid trials for each condition exceeded 80 per participant.

Although the experimental task involved four outcome categories, the primary objective of this study was to examine the differences between near-miss loss, full loss and full win. Consequently, only EEG data elicited by these three outcome types (near-miss loss, full loss, and full win) were separately averaged.

**Figure 2:**
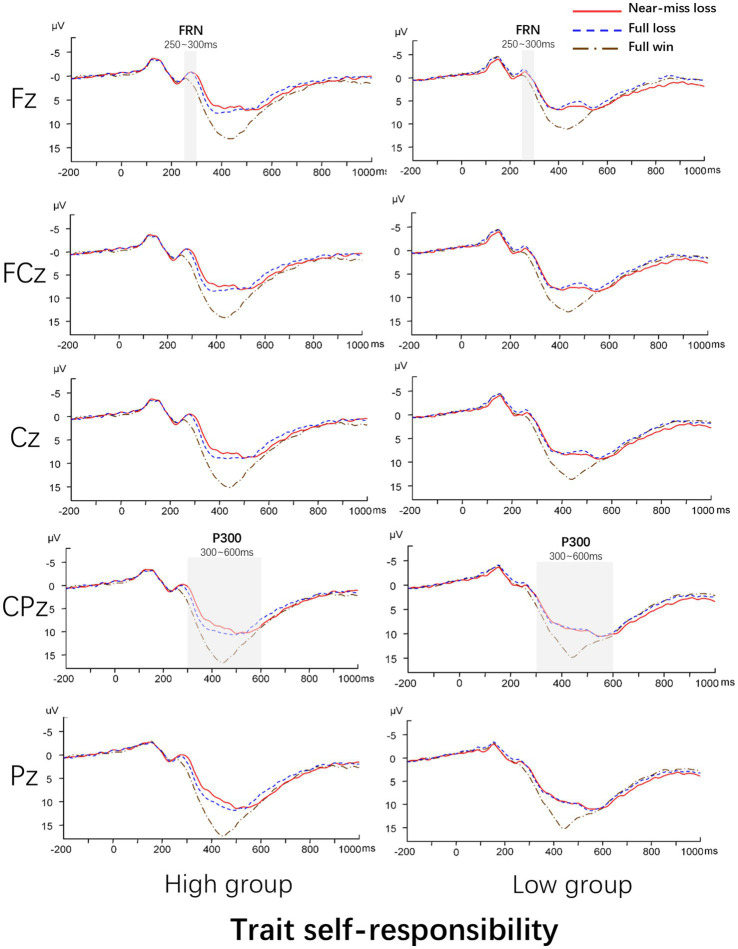
Grand average ERPs at midline electrodes. The grey shaded vertical bars indicate the time windows selected for the mean amplitude analysis of the FRN (250–300 ms) and P300 (300–600 ms).

Based on the grand average waveforms in Figure 2 and previous studies (Gehring and Willoughby, 2002; Hajcak et al., 2005; Yeung et al., 2005; Suo et al., 2009; Yue et al., 2011), the mean amplitude of the FRN was analyzed at electrode site Fz with a 250–300 ms time window, while the mean amplitude of the P300 was analyzed at electrode site CPz with a 300–600 ms time window.

### 2.4 Statistical analysis

The experiment employed a 2 (Trait self-responsibility: High-TSR group, Low-TSR group) × 3 (Outcome type: near-miss loss, full-loss, full win) mixed design. Trait self-responsibility was a between-subjects variable with two factor, and outcome type was a within-subjects factor. The dependent variables were the behavioral ratings, FRN, and P300 mean amplitudes.

*A priori* power analysis was conducted using G*Power 3.1 (Faul et al., 2007) to determine the sufficient sample size for detecting a significant interaction effect in a 3 × 2 mixed-design ANOVA. Based on a medium effect size (*f* = 0.25), a significance level (*α*) of 0.05, and a statistical power (1-*β*) of 0.80, assuming a correlation of 0.50 among repeated measures, the analysis indicated that a minimum total sample size of 28 participants (14 per group) was required to achieve statistical power. Furthermore, a post-hoc sensitivity analysis was performed to determine the minimal detectable effect size given the final sample size of 30. Results indicated that with *α* = 0.05 and 1-*β* = 0.80, the study was sensitive enough to detect a minimum effect size of *f* = 0.24.

For behavioral rating data, we conducted five separate 3 (Outcome type) × 2 (Trait self-responsibility) mixed ANOVAs. For ERP data, we also conducted two 3 (Outcome type) × 2 (Trait self-responsibility) mixed ANOVAs on the mean amplitudes of the FRN and P300. All statistical analyses were performed using SPSS 21.0. The Greenhouse–Geisser correction was applied when the assumption of sphericity was violated. Post-hoc tests with Bonferroni correction were used for follow-up comparisons of significant effects.

## 3 Results

### 3.1 Behavioral rating data

**Figure 3:**
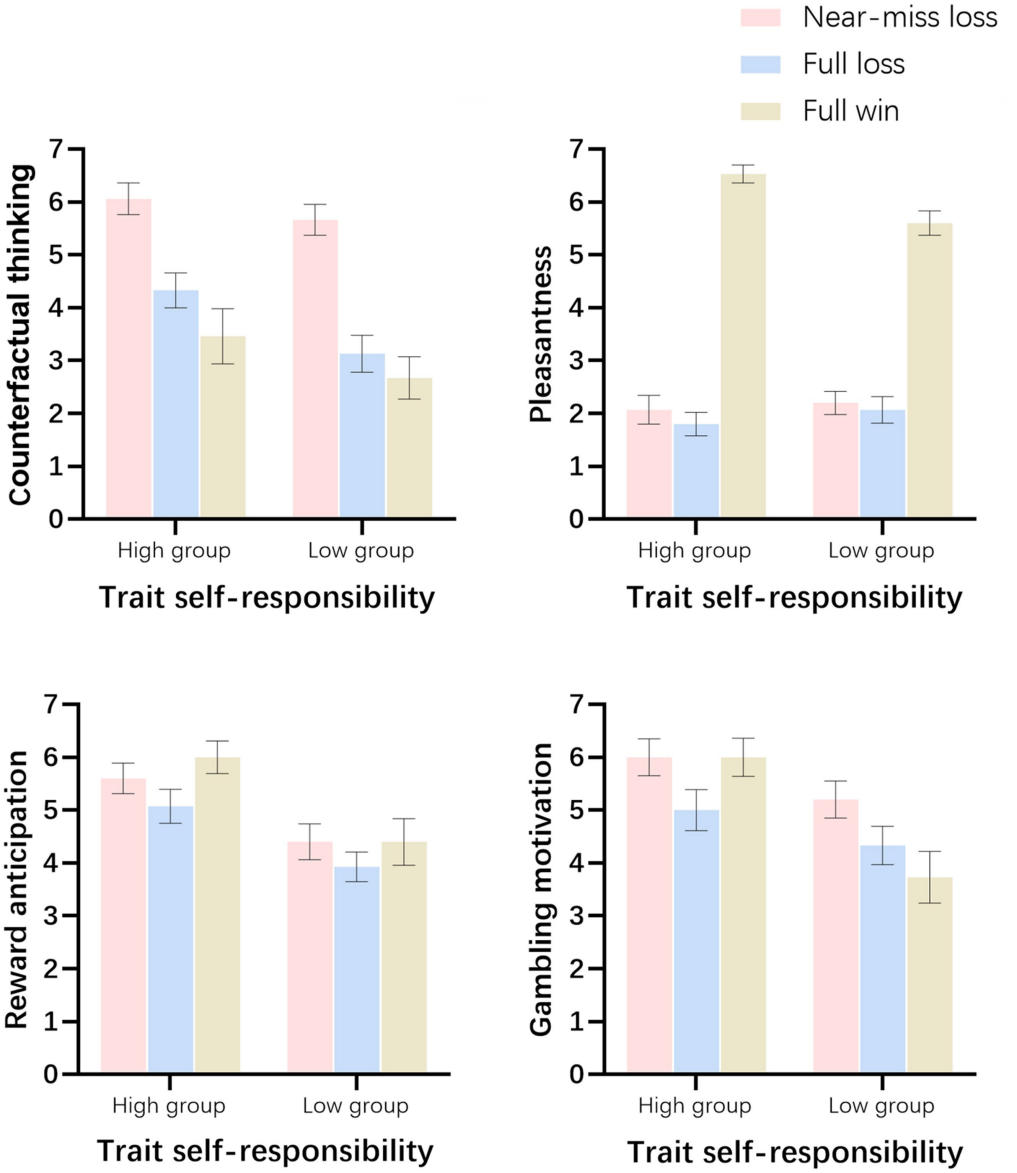
Subjective ratings of gambling outcomes modulated by trait self-responsibility. Error bars represent standard errors.

The behavioral ratings for the different outcomes are shown in Table 1 and illustrated in Figure 3.

**Table 1:** Descriptive statistics of behavioral ratings across outcome types for high and low trait self-responsibility groups.

Variable	Trait self-responsibility	Near-miss loss		Full loss		Full win	
		*M*	*SD*	*M*	*SD*	*M*	*SD*
Perceived responsibility	High	5.47	0.83	5.33	1.17	5.53	1.60
	Low	3.73	1.39	3.27	1.67	4.53	1.71
Counterfactual thinking	High	6.06	1.16	4.33	1.29	3.46	2.03
	Low	5.66	1.11	3.13	1.35	2.67	1.54
Pleasantness	High	2.07	1.03	1.80	0.86	6.53	0.64
	Low	2.20	0.86	2.07	0.96	5.60	0.91
Reward anticipation for the next trial	High	5.60	1.12	5.07	1.22	6.00	1.20
	Low	4.40	1.30	3.93	1.10	4.40	1.72
Gambling motivation	High	6.00	1.36	5.00	1.51	6.00	1.41
	Low	5.20	1.37	4.33	1.40	3.73	1.91

#### 3.1.1 Perceived responsibility

The interaction between trait self-responsibility and outcome type was not significant [*F*(2,56) = 1.93, *p* = 0.16]. However, the main effect of trait self-responsibility was significant [*F*(1,28) = 14.98, *p* = 0.001, *η*^2^ = 0.35]. The High-TSR group reported significantly stronger perceived responsibility across all outcome types compared to the Low-TSR group (see Table 1). This result further validates the effectiveness of the participant grouping.

#### 3.1.2 Counterfactual thinking

The statistical analysis revealed no significant interaction between trait self-responsibility and outcome type [*F*(2,56) = 0.91, *p* = 0.41]. The main effect of trait self-responsibility was significant [*F*(1,28) = 3.93, *p* = 0.05, *η*^2^ = 0.12], with the High-TSR group reporting significantly more intense counterfactual thinking than the Low-TSR group (*M*_high group_ = 4.62 ± 0.28, *M*_low group_ = 3.82 ± 0.28). The main effect of outcome type was also significant [*F*(2,56) = 48.62, *p* < 0.001, *η*^2^ = 0.64]. Post-hoc tests revealed that near-miss losses elicited significantly stronger counterfactual thinking than both full losses and full wins (all *ps* < 0.001, see Table 1).

#### 3.1.3 Pleasantness

The statistical analysis revealed a significant interaction between trait self-responsibility and outcome type [*F*(2, 56) = 3.46, *p* = 0.043, *η*^2^ = 0.11]. Further simple effects analysis indicated that for the High-TSR group, pleasantness ratings differed significantly across outcomes [*F*(2, 56) = 128.05, *p* < 0.001, *η*^2^ = 0.90], with the highest rating for full wins, followed by near-miss losses, and the lowest for full losses(*M*_near-miss loss_ = 2.07 ± 1.03, *M*_full loss_ = 1.80 ± 0.86, *M*_full win_ = 6.53 ± 0.64). For the Low-TSR group, while full wins elicited the strongest pleasantness, there was no significant difference between near-miss losses and full losses [*F*(2,28) = 75.34, *p* < 0.001, *η*^2^ = 0.84; *M*_near-miss loss_ = 2.20 ± 0.86, *M*_full loss_ = 2.06 ± 0.96, *M*_full win_ = 5.60 ± 0.91] (see Table 1; Figure 3). The main effect of outcome type was significant [*F*(2,56) = 198.64, *p* < 0.001, *η*^2^ = 0.88], while the main effect of trait self-responsibility was marginally significant [*F*(1,28) = 3.65, *p* = 0.066].

#### 3.1.4 Reward anticipation for the next trial

Statistical analysis revealed no significant interaction between trait self-responsibility and outcome type [*F*(2,56) = 0.35, *p* = 0.61]. The main effect of trait self-responsibility was significant [*F*(1,28) = 16.72, *p* < 0.001, *η*^2^ = 0.37]. The High-TSR group reported significantly stronger reward anticipation for the next trial compared to the Low-TSR group (*M*_high group_ = 5.56 ± 0.23, *M*_low group_ = 4.24 ± 0.23). The main effect of outcome type was marginally significant [*F*(2,56) = 2.88, *p* = 0.065, *η*^2^ = 0.093]. Reward anticipation elicited by near-miss losses and full wins was higher than that elicited by full losses (*M*_near-miss loss_ = 5.00 ± 1.34, *M*_full loss_ = 4.50 ± 1.28, *M*_full win_ = 5.20 ± 1.67).

#### 3.1.5 Gambling motivation

The analysis revealed a significant interaction between trait self-responsibility and outcome type [*F*(2,56) = 5.38, *p* = 0.009, *η*^2^ = 0.16]. Simple effects analysis indicated that for the High-TSR group, gambling motivation elicited by near-miss losses and full wins was significantly higher than that elicited by full losses [*F*(2,28) = 4.87, *p* = 0.022, *η*^2^ = 0.26; *M*_full win_ = 6.00 ± 1.41, *M*_near-miss loss_ = 6.00 ± 1.36, *M*_full loss_ = 4.60 ± 1.30]. In contrast, for the Low-TSR group, gambling motivation was highest following near-miss losses, followed by full losses, and lowest following full wins [*F*(2, 28) = 6.28, *p* = 0.006, *η*^2^ = 0.31; *M*_near-miss loss_ = 5.20 ± 1.37, *M*_full loss_ = 4.33 ± 1.40, *M*_full win_ = 3.53 ± 1.68] (see Table 1; Figure 3). The main effects of both trait self-responsibility [*F*(1,28) = 12.82, *p* = 0.001, *η*^2^ = 0.31] and outcome type [*F*(2, 56) = 5.63, *p* = 0.008, *η*^2^ = 0.17] were also significant.

### 3.2 ERP data

#### 3.2.1 FRN

The interaction effect was not significant [*F*(2,56) = 1.02, *p* = 0.37], nor was the main effect of trait self-responsibility [*F*(1,28) = 2.02, *p* = 0.17]. The main effect of outcome type was significant [*F*(2, 56) = 13.95, *p* < 0.001, *η*^2^ = 0.33]. Post-hoc tests revealed no significant difference between the FRN amplitudes elicited by near-miss losses and full losses. However, both loss types elicited significantly larger (more negative) FRN amplitude than full wins (*M*_near-miss loss_ = −0.21 ± 0.61 μV, *M*_full loss_ = −0.51 ± 0.61 μV, *M*_full win_ = 0.81 ± 0.62 μV). This indicates that the near-miss effect was not observed in the FRN component for either groups (see Table 2; Figure 2).

**Table 2:** Descriptive statistics of ERP mean amplitudes (in μV).

ERP component (Site)	Trait self-responsibility	Near-miss loss		Full loss		Full win	
		*M*	*SD*	*M*	*SD*	*M*	*SD*
FRN (Fz)	High	0.55	2.50	0.55	2.34	1.52	2.71
	Low	−0.97	4.01	−1.57	4.12	0.09	3.98
P300 (CPz)	High	6.29	4.80	7.87	5.22	11.84	5.79
	Low	7.10	5.78	7.25	6.53	10.59	7.22

#### 3.2.2 P300

A significant interaction [*F*(2,56) = 3.62, *p* = 0.038, *η*^2^ = 0.087]. Simple effects analysis demonstrated that for the High-TSR group, P300 differed significantly across outcomes [*F*(2,28) = 68.32, *p* < 0.001, *η*^2^ = 0.78], with the smallest amplitude for near-miss losses, followed by full losses, and the largest for full wins (*M*_near-miss loss_ = 6.29 ± 4.80 μV, *M*_full loss_ = 7.88 ± 5.22 μV, Mfull win = 11.84 ± 5.79 μV, all pairwise comparisons *p* < 0.01). In contrast, for the Low-TSR group, P300 amplitudes also differed [*F*(2,28) = 20.69, *p* < 0.001, *η*^2^ = 0.52], but the amplitudes elicited by near-miss losses and full losses were statistically equivalent and both smaller than that for full wins (*M*_near-miss loss_ = 7.10 ± 5.78 μV, *M*_full loss_ = 7.25 ± 6.53 μV, *M*_full win_ = 10.59 ± 7.22 μV).

These findings suggest that the near-miss effect on the P300 is significantly modulated by trait self-responsibility. Specifically, higher trait self-responsibility is associated with a more pronounced attenuation of the P300 specifically for near-miss losses relative to full losses (see Table 2; Figure 2).

## 4 Discussion

The present study aimed to investigate whether trait self-responsibility modulates the behavioral and neural responses to near-miss loss in gambling. By comparing high and low self-responsibility groups, we found that this personality trait significantly influenced subjective experiences and neural processing of near-miss loss, partially supporting our hypotheses.

### 4.1 Behavioral results

#### 4.1.1 Counterfactual thinking

Our results indicated that near-miss loss induced higher counterfactual thinking than full loss and full win. This is consistent with previous study (Yin et al., 2024), further validates the established understanding of near-miss loss that promotes thoughts about what might have been (Kahneman and Varey, 1990). Moreover, the high trait self-responsibility group demonstrated an overall elevation in counterfactual thinking. This indicates that an individual’s perception of responsibility for outcomes significantly influences the generation of counterfactual thoughts. This result provides empirical support for the functional theory of counterfactual thinking (Roese, 1997), which posits a strong link between causal attribution and mental simulation. By showing that high trait self-responsibility (characterized by an internal locus of control) elevates baseline counterfactual thinking, we confirm that perceived agency is a prerequisite for the intensive generation of self-referential counterfactuals.

#### 4.1.2 Pleasantness

Analysis of pleasantness ratings revealed a significant interaction between trait self-responsibility and outcome type, indicating that the two groups interpreted the “closeness” of the loss differently. For the low trait self-responsibility group, pleasantness ratings for near-miss loss did not differ significantly from full losses. This pattern suggests a binary interpretation of gambling outcomes: without a perceived causal link between personal action and the result (i.e., attributing outcomes to luck), a miss is simply processed as a failure, regardless of its proximity to the winning. In contrast, the high trait self-responsibility group rated near-miss loss as more pleasant (or less unpleasant) than full losses. From the perspective of illusion of control (Langer, 1975), individuals with high perceived agency may process near-miss loss not merely as failures, but as a signal of skill acquisition (Clark et al., 2009). For these individuals, the proximity to the win may validate their strategy or effort, providing a subtle form of cognitive reinforcement that mitigates the negative affect typically associated with losing.

#### 4.1.3 Reward anticipation for the next trial

Our analysis of reward anticipation revealed a main effect of trait self-responsibility, with the high trait self-responsibility group reporting significantly stronger anticipation of winning for the subsequent trial. This finding elucidates the motivational mechanism linking perceived agency to gambling persistence. According to the illusion of control theory (Langer, 1975), individuals who perceive a skill element in chance events tend to overestimate their probability of success. For the high trait self-responsibility group, the act of stopping the wheel likely fostered a sense of causal agency. Consequently, these individuals may interpret losses not as bad luck, but as temporary performance errors that can be “corrected” in the next trial, thereby sustaining a chronically high level of reward anticipation (Wohl and Enzle, 2002). Regarding winning, these individuals likely interpret success as validation of their skill. This belief can trigger the “hot hand fallacy,” which indicates the conviction that a successful outcome increases the probability of subsequent success due to perceived momentum (Ayton and Fischer, 2004).

#### 4.1.4 Gambling motivation

The analysis of gambling motivation yielded a significant interaction, highlighting distinct motivational drivers for high and low self-responsibility groups. For the high trait self-responsibility group, gambling motivation was equally high following full win and near-miss loss, both of which were significantly higher than full loss. This pattern also mirrors the “skill signal” hypothesis (Clark et al., 2009). Individuals with high perceived agency likely interpret full win as validation of their mastery and near-miss loss as indicators of acquiring the skill. Consequently, both outcomes serve as positive reinforcement. This suggests that for high-responsibility individuals, the motivation to gamble is driven by the pursuit of competence, making them susceptible to prolonged play regardless of whether they are actual winning or nearly winning.

Importantly, the significantly higher baseline motivation in the high group confirms that trait self-responsibility acts as a catalyst for gambling persistence. While low trait self-responsibility individuals may “quit while ahead” (as seen in their lower motivation after wins), high-responsibility individuals are trapped in a cycle where both win and near-miss loss fuel the desire to continue.

### 4.2 ERP results

#### 4.2.1 FRN

Contrary to our hypothesis, trait self-responsibility did not modulate the FRN. Both groups showed a similar FRN effect: near-miss loss and full loss elicited more negative amplitudes than full win, with no significant difference between the two loss types. This resembles some prior studies (Habib and Dixon, 2010; Qi et al., 2011), which suggest that the FRN, as an early, semi-automatic index of prediction error generated in the ACC (Holroyd and Coles, 2002), primarily codes the binary valence of the outcome (win vs. loss). At this early stage of processing, the brain appears to classify a near-miss simply as “not a win,” regardless of the individual’s trait self-responsibility. This finding implies that the modulatory effect of trait self-responsibility occurs at later, more cognitive stages of processing.

#### 4.2.2 P300

The results revealed a significant interaction, which validates the moderating role of trait self-responsibility in the near-miss effect. For the High-TSR group, near-miss losses elicited smaller P300 amplitude than full losses.

This finding seems to present a paradox. The High-TSR group found near-miss loss to be behaviorally motivating, yet showed the lowest P300 amplitude. We propose that within the domain of non-reward outcomes, this attenuation is driven by subjective expectancy consistency (Polich, 2007; Yin et al., 2024).

It is important to note that near-miss loss and full loss were objectively equiprobable (96 trials each). Thus, the difference in P300 cannot be attributed to the rarity of the events (frequency effect). Instead, it reflects the participants’ internal model. High-TSR individuals, characterized by a strong illusion of control, likely form an explicit expectancy of performing well. Consequently, near-miss was perceived as consistent with their expectation of skill acquisition (“*I am getting close*”), resulting in a reduced neural “surprise” signal (attenuated P300, Polich, 2007). In contrast, full loss violated this expectancy of control, representing a discrepancy that requires greater attentional updating, thereby eliciting a larger P300 compared to near-miss loss.

## 5 Conclusion and limitations

In summary, the present study provides the first empirical evidence that trait self-responsibility serves as a critical moderator of the near-miss effect, fundamentally altering both subjective experience and neural processing.

Behaviorally, the two groups exhibited distinct motivational and affective profiles. High trait self-responsibility individuals rated near-misses as significantly more pleasant than full losses and maintained maximal gambling motivation following both wins and near-miss losses. Furthermore, they showed a chronically elevated baseline of counterfactual thinking and reward anticipation, reflecting a sustained “illusion of control.” In contrast, low trait self-responsibility individuals did not differentiate the pleasantness of near-miss losses from full losses and reported their lowest gambling motivation following full wins.

Neurally, this modulation occurs specifically at the late cognitive stage rather than the early valuation stage. The FRN was insensitive to trait self-responsibility, reflecting a binary “win vs. non-win” evaluation common to all participants. However, the P300 revealed a significant interaction: high trait self-responsibility individuals exhibited attenuated P300 amplitudes specifically for near-miss losses.

Several limitations of the present study should be acknowledged. First, the sample consisted of healthy university students with a relatively small sample size (*N* = 30). Although the sensitivity analysis confirmed the study’s power to detect medium effect sizes (*f* = 0.24), the small sample size may limit the generalization of the findings and the ability to detect smaller effects. Second, the study employed a laboratory-based gambling task with relatively low monetary stakes. While this design ensures experimental control, it may not fully capture the intense physiological arousal and “loss of control” experienced in real-world high-stakes gambling scenarios. Third, trait self-responsibility was assessed via a self-report questionnaire, which, despite its validity, may be subject to social desirability bias. Future research should consider employing larger, more diverse samples and incorporating behavioral proxies of agency or experimental manipulations of control to further validate the causal link between responsibility and the near-miss effect in distinct neural stages.

## Data Availability

The raw data supporting the conclusions of this article will be made available by the authors, without undue reservation.
